# Papillary Thyroid Carcinoma and Body Mass Index: The Role of Immune System in Tumor Microenvironment

**DOI:** 10.3390/ijms26178290

**Published:** 2025-08-26

**Authors:** Rebecca Sparavelli, Riccardo Giannini, Francesca Signorini, Gabriele Materazzi, Alessio Basolo, Ferruccio Santini, Clara Ugolini

**Affiliations:** 1 Department of Surgical, Medical, Molecular Pathology and Critical Area, University of Pisa, 56100 Pisa, Italy; 2 Endocrine Surgery Unit, Department of Surgical, Medical and Molecular Pathology and Critical Area, Pisa University Hospital, 56126 Pisa, Italy; 3 Obesity and Lipodystrophy Center, Endocrinology Unit, Pisa University Hospital, 56124 Pisa, Italy

**Keywords:** PTC, TME, immune system, BMI, thyroid carcinoma, gene expression analysis, ARG2, HGMB1

## Abstract

Papillary thyroid cancer (PTC) is linked to obesity, but the biological mechanisms that may explain this connection have been only partially described. Potential factors that combine overweight/obesity with this cancer should be searched for in the immune pathways and chronic inflammation onset. In this study, we evaluated the role of the immune system in patients affected by PTC and stratified them according to Body Mass Index (BMI). An analysis of the expression profiles of >700 immune-related genes was performed in 36 PTCs, subdivided into four categories: underweight (A), normal weight (B), overweight (C), and subjects living with obesity (D). B was considered a reference category. In our study, the immune microenvironment of PTCs did not seem strongly influenced by BMI. However, based on the interaction from in silico protein–protein analysis, we found that the dysregulation profiles of groups A or D were similar as concerns pathways involved in T-cell differentiation, macrophage activation, regulation of the cell cycle, and senescence processes. Furthermore, we found significant downregulation of *HMGB1* in the A and D categories, with upregulation of *ARG2* in the D category. Although further studies are necessary, these genes may provide an opportunity to better understand immunometabolism in thyroid cancer.

## 1. Introduction

Differentiated thyroid cancer (DTC), especially papillary thyroid carcinoma (PTC), is the most common endocrine malignancy, accounting for 96.0% of total new endocrine cancers and 66.8% of deaths due to endocrine cancers [1]. However, some variants of PTC have been associated with an increased risk of recurrent disease and aggressive behavior [2,3]. Many exogenous factors, such as obesity and insulin resistance, could be implicated in the pathogenesis of thyroid cancer [4,5,6]. Obesity is a low-grade chronic inflammatory state characterized by an increase in systemic markers of inflammation with non-specific activation of the immune system [7,8,9]. Excess adipose tissue, characteristic of overweight and obesity, significantly alters the endocrine functions of adipose tissue, contributing to the development of various obesity-related malignancies, including those of the endocrine system such as breast, endometrial, ovarian, prostate, and thyroid cancers [10]. This alteration is primarily due to the dysregulation of adipokine secretion, insulin resistance, and chronic low-grade inflammation associated with increased fat mass [10]. Growing evidence suggests that the innate immune cells (macrophages, neutrophils, dendritic cells, innate lymphoid cells, myeloid-derived suppressor cells, and natural killer cells (NK)) and the adaptive immune cells (T-cells and B-cells) contribute to tumor progression when present in the tumor microenvironment (TME) [11,12]. The TME is the area around a tumor, and it has a potential role in tumor progression and response to treatment, through interactions and communications with tumor cells [13]. The immune microenvironment of thyroid cancer includes many immune cells: T-cells, NK cells, mast cells, tumor-associated macrophages, dendritic cells, myeloid-derived suppressor cells, and secreted immune factors such as chemokines [14,15]. In the TME, these immune cells play a crucial role in the initiation and progression of thyroid cancer, affecting various aspects of tumor behavior, such as metabolic conditions. Although a high BMI can increase the risk of PTCs, molecular mechanisms linking excessive adiposity with the development of cancer are complex and still not completely known.

The aim of this study is to molecularly characterize the TME in DTC patients stratified by BMI categories, thereby enhancing our understanding of how BMI modulates thyroid cancer.

## 2. Results

### 2.1. Sample Features

From a total of 238 PTCs, 36 patients were selected. Specifically, six patients were classified as “underweight” (A), sixteen as “normal weight” (B), eight as “overweight” (C) and six as “obesity” (D). All demographic data are summarized in Table A1, with other clinical information.

### 2.2. Normalization and Gene Expression Data

**Figure A1:**
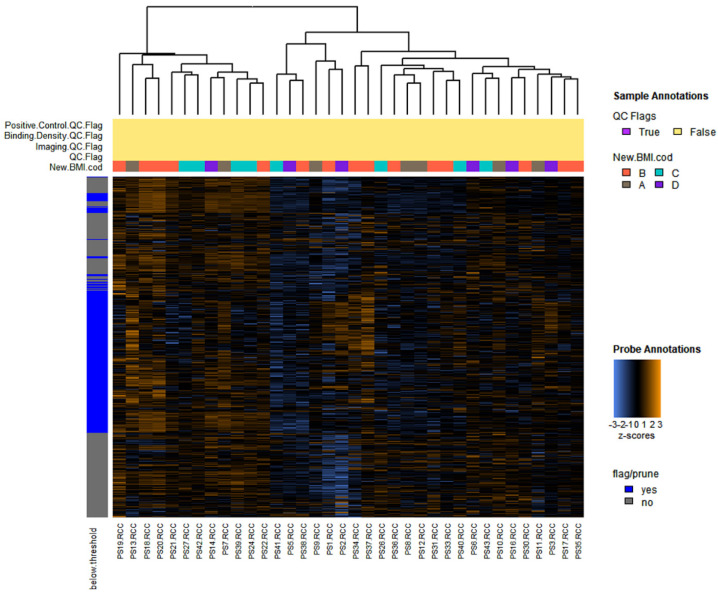
Heatmap of the mRNA-normalized data, scaled to give all genes equal variance and generated via unsupervised clustering. The orange color indicates high expression, while blue denotes low expression. “A” represents “underweight”, “B” represents “normal weight”, “C” represents “overweight”, and “D” represents “obesity”.

The housekeeping genes selected for the normalization of the experiment presented a steady expression level in all the studied samples. No samples were excluded after data normalization. Analysis of gene expression data showed two different gene expression profiles related to PTC samples, apparently not associated with BMI categories. Figure A1 presents the heatmap of the normalized mRNA data, scaled to equalize gene variance, and generated through unsupervised clustering.

### 2.3. Differential Analysis for Gene Functional Groups

**Figure 1:**
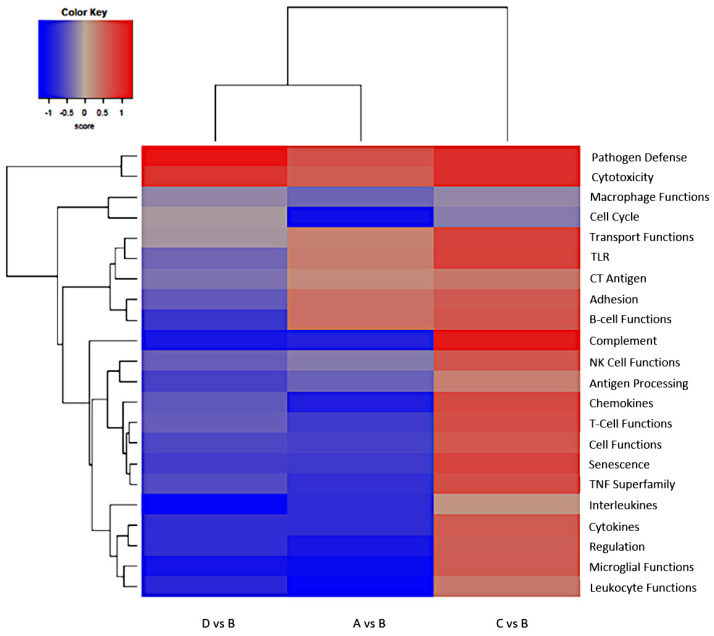
Heatmap displaying each sample’s directed global significance scores. Directed global significance statistics measure the extent to which a gene set’s genes are up- or downregulated with the variable. Red denotes gene sets whose genes exhibit extensive overexpression with the covariate; blue denotes gene sets with extensive underexpression.

In this analysis, 22 gene sets were considered, and all data are shown in Figure 1. Overweight samples (C), compared with normal weight samples (B), exhibited upregulation of twenty-one gene sets and no change in one gene set. Underweight samples (A) showed thirteen upregulated gene sets, five downregulated ones, and four neutral ones. Obesity samples (D) displayed upregulation of fourteen gene sets, downregulation of three gene sets, and five neutral results. When compared to B samples, both A and D categories exhibited similar gene expression profiles, particularly with regard to functional groups. Specifically, they demonstrated comparable differential expression of genes involved in pathogen defense processes, such as the macrophage activation pathway, leukocyte function, cell cycle regulation, senescence, T-cell function, and cytokine release.

### 2.4. Differential Analysis for Single Genes

For this analysis, a comparison was made between the categories of interest and group B.

**Figure 2:**
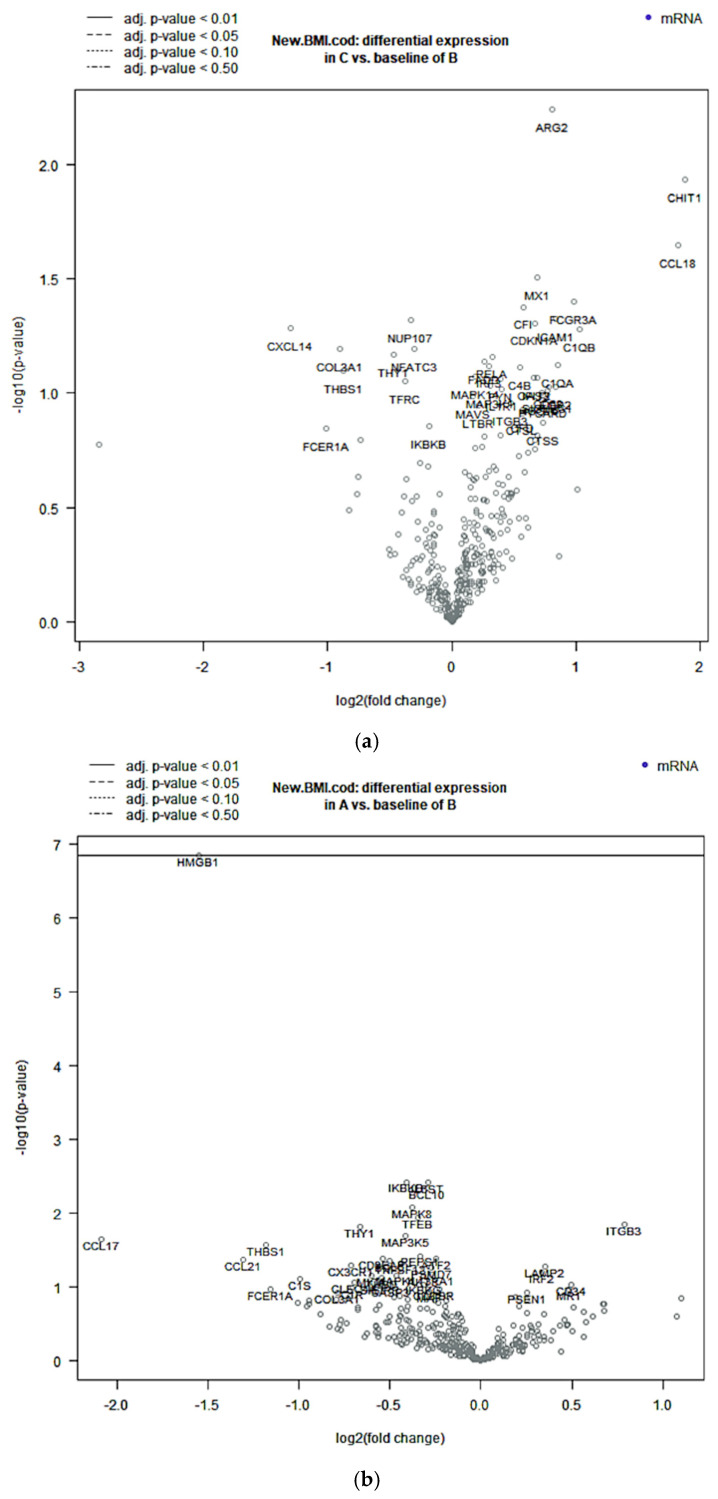

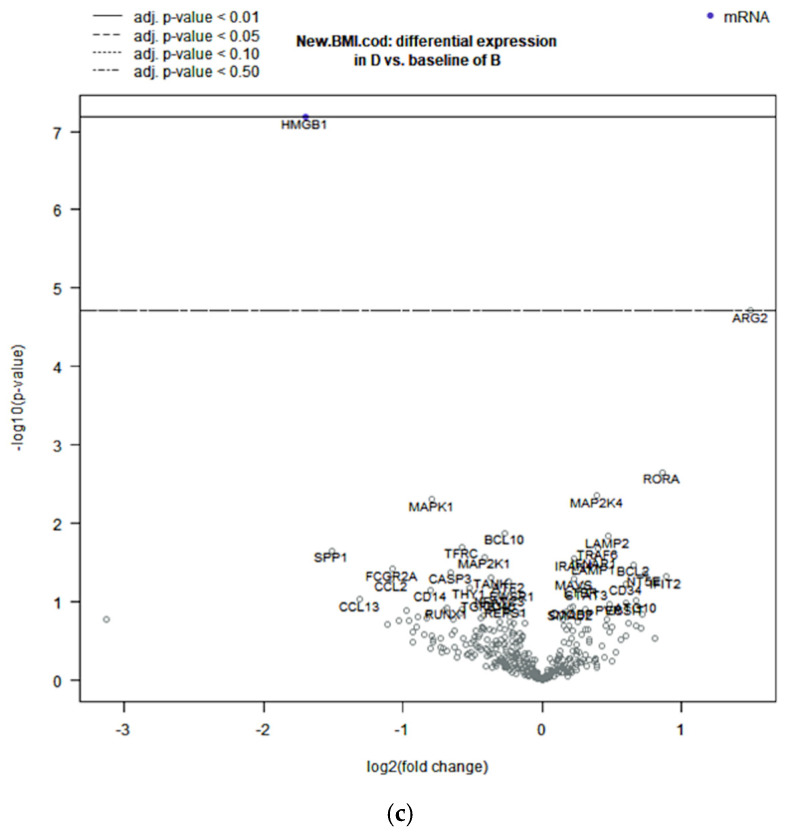
Volcano plots displaying each gene’s −log10 (*p*-value BY) and log2 fold change with the selected covariate. Highly statistically significant genes fall at the top of the plot, above the continuous horizontal lines, and highly differentially expressed genes fall to either side. Horizontal lines indicate various *p*-value thresholds: the continuous horizontal line indicates a *p*-value thresholds <0.01, while a dotted line an higher *p*-value threshold values, as we can see in the legend above the figure. Genes are colored if the resulting *p*-value is below the given *p*-value threshold. (**a**) Overweight samples (C) do not show statistically significant differential gene expression. (**b**) Underweight samples (A) show five statistically significant downregulated genes and only one statistically significant downregulated gene. (**c**) Obesity samples show four statistically significant upregulated genes and five statistically significant downregulated genes.

Compared with B samples, A samples showed statistically significant deregulation in six genes: *CCL17*, *CCL21*, *HMGB1*, *ITGB3*, *THBS1*, and *THY1.* We observed downregulation of *HMGB1*, *CCL17*, *CCL21*, *THBS1*, and *THY1* and upregulation of *ITGB3*. One of these genes, *HMGB1*, was statistically significantly downregulated after application of BY correction (Figure 2b). C samples showed statistically significant upregulation of six genes: *ARG2*, *CCL18*, *CHIT1*, *ICAM1*, *FCGR3A*, and *MX1* (Figure 2a). Finally, D samples showed statistically significant deregulation of nine genes: *ARG2*, *BCL2*, *CASP3*, *FCGR2A*, *HMGB1*, *MAPK1*, *NT5E*, *RORA*, and *SPP1*. We observed downregulation of *HMGB1*, *CASP3*, *MAPK1*, and *SPP1* and upregulation of *ARG2*, *BCL2*, *NT5E*, and *RORA*. Two of these genes, *HMGB1* and *ARG2*, were statistically significant after application of BY correction (Figure 2c). Differentiated genes are visualized in the specific Volcano plot, a graph displaying each gene’s −log10 BY-*p* value and log2 fold change for the specific categories of BMI samples compared to the normal weight category. A complete list of the deregulated genes between our categories of interest and B samples can be found in Supplementary Table S1.

### 2.5. Functional Clustering Analysis

**Figure A2:**
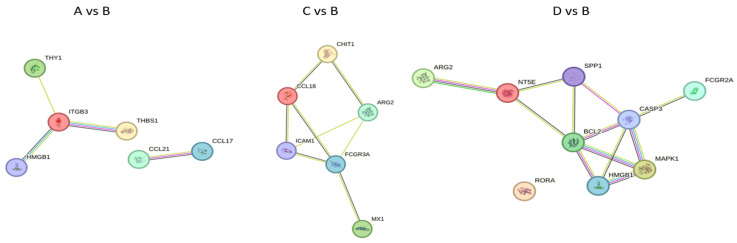
STRING protein interactions of A, C, and D categories compared to B. The edges represent specific and significant protein-protein associations, not necessarily physically connected to each other. Different colors indicate known or predicted interactions. “A” represents “underweight”, “B” represents “normal weight”, “C” represents “overweight”, and “D” represents “obesity”.

Based on interactions retrieved from STRING, protein networks were constructed for the BMI categories. Among the six differentially expressed genes, three (*HMGB1*, *ITGB3*, and *THBS1*) emerged as key interaction nodes. These genes were enriched in pathways involved in positive regulation of leucocyte migration, cell adhesion and cell migration; apoptotic cell clearance; and lipid transport (with an FDR < 0.05). C network showed six nodes and eight edges, and among the six differentially expressed genes, two (*CCL18* and *CHIT1*) stood out as the most relevant interactions nodes. These genes were enriched in pathways involved in immune response as macrophage activation pathways (with an FDR < 0.05). Regarding the D category, nine proteins and twelve edges were included in the network; among the nine differentially expressed genes, four genes (*HMGB1*, *BCL2*, *CASP3*, and *MAPK1*) stood out as the most relevant interaction nodes and were enriched in pathways involved in response to lipids, regulation of leucocyte proliferation, apoptotic processes, and response to organic cyclic compounds (with an FDR < 0.05). These data are summarized in Figure A2.

## 3. Discussion

In the last few years, many studies and meta-analyses have investigated the relationship between obesity and the development of thyroid cancer. Future research directions may also be highlighted [4,8,13,16]. Particularly, changes in immune status within the TME are closely related to tumor progression [17]. Despite the immune system playing a key role in thyroid carcinogenesis, immune cells, with their soluble factors, may exhibit both pro- and anticancer activity. Therefore, they may be potentially correlated with the higher incidence of thyroid cancer in obesity. Metabolic conditions in the TME are influenced by gradients of nutrients, oxygen levels, tissue vascularization, heterocellular interactions, and systemic metabolism [18]. The TME is a dynamic environment continuously shaped by complex, highly heterogeneous cellular and environmental factors, supporting both intra-tumoral and inter-tumoral heterogeneity [19]. Therefore, it is presumed that the TME is affected by and influences tumor characteristics differently, depending on potential metabolic conditions. Rodriguez PC et al. showed that, during tumor progression, cancer cells increase expression of amino acids transporters, reducing amino acids in the TME, hindering expansion, differentiation, and function in tumor-infiltrating lymphocytes (TILs) [20]. These findings underscore the importance of understanding metabolic interactions in the TME to develop strategies that enhance anti-tumor immunity. Arginine and tryptophan, for example, have a role in the regulation of T-cell survival, proliferation, and activation, becoming a potential target of tumor immunotherapy [21]. Furthermore, the TME influences tumor cell proliferation, invasion and metastasis of cancer cells, and angiogenesis, and it has an important impact on the therapy response [22]. Hypoxia, for example, a relevant factor of the TME, is the key regulator for chemo-/radioresistance in different cancer types [23]. In this study, we aimed to evaluate the function and activity of the immune system in the TME of patients affected by PTC and belonging to different BMI classes. In our study, based on the expression of mRNA, the immune system does not seem to be strongly influenced by BMI. Considering differential analysis for gene functional groups, A and D categories are both characterized by a similar gene expression profile. In detail, they showed similar deregulation of genes involved in pathogen defense processes, such as leukocyte and macrophage activation pathways, T-cell function, cytokines release, regulation of the cell cycle, and senescence processes. They also exhibit similar in silico interaction profiles in the regulation of leukocyte migration and proliferation, lipid transport, and senescence processes. Such gene expression similarities in two extremely different metabolic conditions may be the contribution of additional variables unrelated to BMI. Contrary to many observational studies, recent studies did not find an association between obesity and thyroid cancer [24,25]. Shakar and William have suggested that patients with cancer and a normal BMI have worse outcomes than patients with obesity, outlining the “obesity paradox”, a controversial concept in the context of cancer management [26]. Mukherjee S. et al. showed that inflammation in the TME is associated with survival outcomes in metastatic gastroesophageal adenocarcinoma patients, regardless of obesity [27]. In the same way, Zeigler-Johnson C. et al. reported that the number of lymphocytes and macrophages in the TME of prostate cancer patients did not differ by obesity status [28]. Considering the differential expression of genes, A and D samples showed downregulation of several genes of high-mobility group box 1 (HMGB1), a highly conservative nucleoprotein belonging to a group of non-histone chromatin-associated proteins, which plays a significant role in many diseases, especially inflammatory diseases and cancer [29]. The expression of HMGB1 in thyroid cancer cells is involved in autophagy processes by regulating NIS degradation and iodide uptake of thyroid cancer cells; for this reason, it is deepened in the context of radioresistance [30]. Although the function of HMGB1 has been evaluated across different types of cancer, few studies have investigated its role across different BMI classes. In addition, D samples showed upregulation of arginase 2 (ARG2). Interestingly, ARG2, the extrahepatic mitochondrial enzyme that catabolizes arginine into ornithine and urea, is induced upon obesity. Obesity or AKT activation might further exaggerate this process, generating excess levels of nitrogen and creating a dependency on ARG2 [31]. ARG2 appears to serve tissue-specific and context-dependent functions in energy homeostasis. Upregulated arginase expression is associated with pathological processes such as cardiovascular, immune-mediated, tumorigenic conditions and neurodegenerative disorders [32]. The role of ARG2 has been investigated in cardiovascular disease [33], fibroblasts senescence [34], melanosomes senescence [35], nonalcoholic fatty liver disease (NAFLD) associates with obesity and type 2 diabetes [36], pancreatic cancer [31], and non-small-cell lung cancer [37].

A recent study showed that the induction of ARG2 would promote the growth of pancreatic ductal adenocarcinoma, by directing metabolic nitrogen flow into the urea cycle; this condition could be increased by obesity and metabolic syndrome, characterized by an overabundance of nutrients [31]. Therefore, arginine deprivation is becoming a novel and promising clinical strategy for metabolism-based cancer therapy [38].

However, our study has several limitations, including its retrospective nature and small sample size. Incorporating direct measures of body composition and key metabolic parameters—such as waist circumference, glycemic control, and lipid profile—may help provide a more comprehensive assessment of adiposity and metabolic health. Further research with larger cohorts is necessary to validate these findings and elucidate the role of the immune microenvironment in the relationship between obesity and thyroid cancer incidence.

## 4. Materials and Methods

### 4.1. Tissue Selection and Histologic Revision

A cohort of 238 patients diagnosed with papillary thyroid carcinoma (PTC) was retrospectively analyzed. These patients were referred to the Endocrinology Unit at Azienda Ospedaliero-Universitaria Pisana, Pisa, Italy, between 2010 and 2012. Body Mass Index (BMI) was calculated as weight (kg) divided by height squared (m^2^). According to the World Health Organization (WHO) classification, patients were categorized as follows:

- Underweight: BMI < 18.5 kg/m^2^;
- Normal weight: BMI 18.5–24.9 kg/m^2^;
- Overweight: BMI 25.0–29.9 kg/m^2^;
- Obesity: BMI ≥ 30.0 kg/m^2^.

Despite BMI being an imperfect surrogate for adiposity, direct measures of body composition and metabolic parameters were not available. Conforming to pathological criteria, tissue selection and histologic revision of the primary tumor samples [formalin-fixed, paraffin-embedded (FFPE)] were carried out.

Classic PTCs were selected, with tumor characteristics (size of tumor, extrathyroid extension, and tumor–node–metastasis staging system characteristics) as similar as possible, to minimize variables and consider BMI as our topic of interest. The most representative paraffin block of each sample, with tumor areas and the TME, was selected for analysis. The study was conducted anonymously and in compliance with the principles of the Declaration of Helsinki. 

### 4.2. Nucleic Acid Extraction and Purification

For each sample, four unstained sections (5 mm thick) were used for RNA extraction. The unstained sections were deparaffinized with xylene and rehydrated in decreasing-grade ethanol solution. We performed manual microdissection to maximize the amount of tumor cells and the TME. RNA was isolated using the RNeasy FFPE Kit (Qiagen, Hilden, Germany), according to the manufacturer’s instructions. RNA was eluted in 18 µL of RNase-free water. RNA quantity and quality were assessed by means of a spectrophotometer (Xpose Trinean, Gentbrugge, Belgium).

### 4.3. Immune-Related Gene Expression Analysis

We performed analysis of the expression profiles of >700 immune-related genes using the nanoString nCounter PanCancer Immune Profiling Panel (NanoString Technologies, Seattle, WA, USA). In detail, 100 ng of RNA from each sample was hybridized with the nCounter PanCancer Immune Profiling Panel (GX Assay) CodeSet. All the procedures, including mRNA quantification, sample preparation, hybridization, detection, and scanning, were performed following the instruction of manufacturer. We normalized the counts according to the standard protocol. Raw nanoString counts for each mRNA within each experiment were subjected to technical normalization, with the counts obtained for positive-control probe sets before biological normalization using the 40 reference genes included in the CodeSet. The normalized data were log2-transformed and then used as input for differential expression analysis. The data were filtered to exclude relatively invariant features and features below the detection threshold (defined for each sample by a cutoff value corresponding to twice the SD of the negative control probes plus the means).

### 4.4. Differential Analysis for Gene Functional Groups and for Single Genes

The PanCancer Immune Profiling Advanced Analysis Module (NanoString Technologies) was used to conduct the statistical analysis of data obtained by the nCounter panel analysis. The analysis module grouped the genes into functional immune-related categories. In particular, 22 functional groups of genes were identified, including those involved in transporter functions, the tumor necrosis factor superfamily, macrophage functions, antigen processing, adhesion, regulation, T-cell functions, cytokines, B-cell functions, ILs, toll-like receptors, cytotoxicity, pathogen defense, cancer/testis antigens, complement, natural killer cell functions, chemokines, leukocyte functions, the cell cycle, senescence, and microglial functions. To investigate the differential expression between the categories of samples in this study, the main covariate considered was BMI. To assess differential gene expression across BMI categories, “normal weight” samples were designated as the reference group. Given the extensive number of genes in the CodeSet, the use of raw *p*-values was deemed inappropriate due to multiple testing concerns. Therefore, genes with a fold change >0.5 and an unadjusted *p*-value < 0.05 were considered differentially expressed. Furthermore, differentially expressed genes with a BY-adjusted *p*-value < 0.05 were reported and considered as the most statistically significant differentially expressed genes.

### 4.5. Functional Clustering Analysis

Functional clustering analysis was conducted using the immune-related gene categories identified through the PanCancer Immune Profiling Advanced Analysis Module. This analysis aimed to evaluate gene expression patterns and corresponding potential protein networks across different BMI categories. In silico analysis was performed by STRING (version 11.5), an online tool to explore the predicted protein–protein interactions, including physical and functional associations, through an algorithm used to group the genes into annotation clusters based on pre-computed similarity information (https://string-db.org/) [39]. To perform clustering analysis, a non-parametric test, termed “Aggregate Fold Change”, was chosen. This test worked, for each gene set to be tested, the average of all values provided by the user for the constituent genes. This average was then compared against averages of randomized gene sets of the same size [39]. The *p*-value was adjusted using the false discovery rate (FDR), an estimate of the probability of false positive results.

## 5. Conclusions

The rising incidence of PTC has been associated with the obesity epidemic, yet the biological mechanisms underlying this relationship remain poorly understood. In the current study, although BMI does not significantly impact the immune microenvironment of PTC, HMGB1 is downregulated in underweight individuals and individuals with obesity, and ARG2 is upregulated in the obesity condition, suggesting their involvement in cancer-related processes. These findings highlight the importance of further research into the role of immunometabolism in thyroid cancer.

## Data Availability

The raw data supporting the conclusions of this article will be made available by the authors upon request.

## Supplementary Materials

The supporting information can be downloaded at: https://www.mdpi.com/article/10.3390/ijms26178290/s1.

## Abbreviations

The following abbreviations are used in this manuscript:

ARG2	Arginase 2; extra-hepatic arginase; mitochondrial arginase
BY	Benjamini–Yekutieli
BMI	Body Mass Index
DTCs	Differentiated thyroid cells
ETE	Extrathyroid extension
FDR	False discovery rate
FFPE	Formalin-fixed paraffin-embedded
HGMB1	High-mobility group box 1
PTC	Papillary thyroid cancer
TILs	Tumor-infiltrating lymphocytes
TME	Tumor microenvironment
WHO	World Health Organization
NIS	Sodium/Iodide Symporter

## Appendix A

**Table A1:** Thirty-six PTCs: patient characteristics. Abbreviations: F, female; M, male; BMI, Body Mass Index; ETE, extrathyroid extension.

Sample ID	Sex	BMI	BMI Category	Size Tumor	ETE	Embolism	Multifocality	T (8th ed)	N
PS7	F	10.8	A	1	Yes	No	Unifocal	T1a(s)	Nx
PS8	F	18	A	1.2	Yes	No	Multifocal	pT1b(m)	N1b
PS9	F	18	A	2.2	No	No	Multifocal	pT2(m)	Nx
PS10	M	18.1	A	2.5	No	No	Unifocal	pT2(s)	N1b
PS11	F	18.3	A	2.1	No	No	Unifocal	pT2(s)	N1a
PS12	F	18.3	A	1.5	Yes	No	Unifocal	pT1b(s)	N1a
PS13	F	18.8	B	1.3	No	No	Multifocal	pT1b(m)	Nx
PS33	F	20	B	1.4	No	No	Multifocal	pT1b(m)	Nx
PS34	F	20.2	B	2	No	No	Unifocal	pT1b(s)	N1b
PS35	F	21.1	B	1.3	Yes	No	Multifocal	pT1b(m)	Nx
PS17	F	21.2	B	1.2	Yes	No	Multifocal	pT1b(m)	Nx
PS18	F	21.6	B	1.4	Yes	No	Multifocal	pT1b(m)	N1a + N1b
PS1	F	21.7	B	2.5	No	No	Unifocal	pT2(s)	Nx
PS19	M	22	B	3.5	No	Yes	Unifocal	pT2(s)	N1b
PS36	F	22.3	B	2.8	No	No	Multifocal	pT2(m)	Nx
PS30	F	22.4	B	2	Yes	No	Multifocal	pT1b(m)	N1a + N1b
PS20	F	22.9	B	2.5	Yes	No	Multifocal	pT2(m)	N1a
PS37	F	23.1	B	2.5	No	No	Unifocal	pT2(s)	N1a
PS21	F	24	B	1.3	Yes	No	Multifocal	pT1b(m)	N1a + N1b
PS22	M	24	B	2	Yes	No	Multifocal	pT1b(m)	Nx
PS38	F	24.4	B	1.1	No	No	Multifocal	pT1b(m)	Nx
PS31	F	24.6	B	1.7	No	No	Unifocal	pT1b(s)	Nx
PS24	M	25.1	C	2.2	No	No	Multifocal	pT2(m)	N1a
PS39	F	25.2	C	2	No	No	Unifocal	pT1b(s)	Nx
PS40	F	26.2	C	2	No	No	Unifocal	pT1b(s)	N0
PS41	F	26.9	C	2.3	Yes	No	Unifocal	pT2(s)	Nx
PS42	F	27	C	1.1	No	No	Unifocal	pT1b(s)	Nx
PS26	M	27.9	C	1.8	Yes	No	Unifocal	pT1b(s)	N1a + N1b
PS43	F	28	C	1.6	No	No	Multifocal	pT1b(m)	Nx
PS27	F	28.2	C	1.6	Yes	No	Multifocal	pT1b(m)	Nx
PS16	F	32.9	D	2.8	Yes	No	Unifocal	pT2(s)	N1a
PS14	M	37.2	D	1.7	No	No	Multifocal	pT1b(m)	Nx
PS6	M	37.4	D	1.5	Yes	No	Multifocal	pT1b(m)	Nx
PS5	M	39.2	D	3	No	No	Multifocal	pT2(m)	Nx
PS3	F	45	D	1.5	Yes	No	Unifocal	pT1b(s)	Nx
PS2	F	62.8	D	2	No	No	Multifocal	pT1b(m)	Nx
